# Supplementary material for: Explainable AI-based Alzheimer’s prediction and management using multimodal data
Source: PLoS One. 2023 Nov 16;18(11):e0294253. doi: 10.1371/journal.pone.0294253 (PMC10653516; doi:10.1371/journal.pone.0294253)
Supplement: Attachment — Submitted filename: Response to Reviewers.docx [file pone.0294253.s001.docx]

PONE-D-23-09483

Explainable AI-based Alzheimer’s Prediction and Management Using Multimodal Data

**Reviewer 3**

**Reviewer #3, Concern#1**:

Could the author elaborate the reason why deep learning models are excluded from the analysis? Neural networks can achieve both feature selection and outcome classification at the same time. Actually, other than the two studies mentioned in the manuscript (Buvari et al. and Kumari et al.), other studies also demonstrated the strength of neural networks on capturing data structure and predicting AD with high accuracy. NNs are one of the famous methods applied to AD data. Considering that the authors using a multimodal dataset, I would recommend looking into the hybrid NN, i.e., the multimodal neural network, which joins information from different modalities to perform a prediction task.

**Author Response:**

We appreciate your feedback, thank you. Since shallow learning methods have yielded satisfactory results in our study, we have opted not to delve into deep learning approaches. To enhance the clarity of our work, we have conducted a comprehensive review of recently published literature and incorporated these findings into our manuscript. Several research studies, including those conducted by Yu et al., Lei et al., Lee et al., Kamal et al., El-Sappagh et al., and Zhang et al., have focused on Alzheimer's Disease (AD) prediction using the ADNI dataset, primarily utilizing MRI and neuroimaging data. Additionally, Buvari et al. employed MRI and Freesurfer segmentation data from the OASIS dataset, achieving an accuracy of approximately 70% through the utilization of Neural Networks.

Amrutesh et al. focused on text data from the OASIS dataset, Ali et al. utilized the OASIS-2 dataset for AD prediction, and both Baglat et al. and Kavita et al. made use of MRI data from the OASIS repository. These authors reported that Random Forest proved to be the top-performing classifier for their respective datasets.

In our work, we utilized clinical data, psychological data, and Freesurfer MRI segmentation data from the OASIS repository. Notably, our experimentation with Neural Networks, despite extensive hyperparameter tuning via grid search, yielded an accuracy of only approximately 25%. This performance discrepancy can be attributed to fundamental dissimilarities between our dataset and those used by previous researchers, rendering Neural Networks less effective for our specific task. For your convenience, we have incorporated the relevant revisions in the specified sections of our manuscript, spanning pages 3-5, lines 61-89, 139-152, and 156-160.

**Author Action:**

**Reviewer #3, Concern#2**:

Additionally, it seems like the accuracy of RF is too good to be true. I wonder are all the results displayed in the manuscript from the testing data not training data? And did the authors check for over-fitting issue? It would be nice if the authors add the details of the training process.

**Author Response:** Thank you for pointing out this issue. The results section provides insights into the model's performance through training, testing, and 10-fold cross-validation accuracy. Utilizing cross-validation ensures a more robust evaluation of the model's generalization to unseen data, effectively mitigating overfitting. This is further substantiated by the AUC values, which demonstrate the absence of overfitting concerns. Table 8 on page 18 outlines the specific modifications made. Additionally, the data was partitioned into an 80% training set and a 20% testing set. The 10-fold cross-validation technique was employed to compute cross-validation accuracy, with detailed information available on pages 11-12 and Lines 336-371.

**Author Action:**

**Reviewer #3, Concern#3**: One last small thing is some abbreviations’ full names appear multiple times in the manuscript, such as ADRC, or appear before the full name, such as OASIS.

**Author Response:**

Thank you. The revised manuscript has been carefully checked to remove any typos, and all abbreviations are introduced the first time they are used.

**Author Action:**

***--------------------------------------------------------------------------------------------------------------------------------------------------------------------------***

**Reviewer 4**

**Reviewer #4, Concern#1:**

Limited evaluation: The paper lacks a thorough evaluation and comparison of the proposed method with existing state-of-the-art models or baselines. Have you compared the performance of your proposed method with other state-of-the-art models or baselines? If not, why not? The authors have provided a list of only 9 widely used machine learning models; however, it should be noted that not all of these models perform at a satisfactory level.

**Author Response:** Thank you very much. The performance of the proposed work has been evaluated in comparison to six recent similar studies. The relevant updates can be found on pages 18 and 19, as well as in lines 536-547 and Table 9.

**Author Action:**

**Reviewer #4, Concern#2:**

Lack of implementation details: The paper does not provide sufficient details on the implementation of the proposed method, making it difficult for readers to reproduce the study. Can you provide more details on the implementation of the proposed method, such as the specific parameters used for each machine learning model?

**Author Response:** Thank you very much. The comprehensive implementation procedure of the proposed model is outlined within the manuscript. You can locate the specific revisions on pages 11-12 and in Lines 337-371.

**Author Action:**

**Reviewer #4, Concern#3:**

Alzheimer’s Prediction using AI is a hot topic. To enhance interest to the readers, it is better to give a more extensive review on this topic. There are some representative works, such as:
Morphological Feature Visualization of Alzheimer's Disease via Multidirectional Perception GAN; Predicting clinical scores for Alzheimer's disease based on joint and deep learning;
Tensorizing GAN With High-Order Pooling for Alzheimer's Disease Assessment.

**Author Response:** Thank you. Three recently published papers are reviewed on the above-mentioned topics and added to the manuscript. Changes can be found on page 3 Lines 61-89.
**Author Action:**

**Reviewer #4, Concern#4:**

Given that the paper focuses on explainability and analysis of prediction causes, it would enhance the overall contribution if there were clinical evidence to support the conclusions.

**Author Response:** Thank you. To assess the significance of the conclusion that Memory, Sumbox, Judgment, Homehobb, and Orient are the most crucial features in predicting the five class outcomes, we conducted t-tests and calculated the corresponding p-values. For all classes, the p-values associated with these features were found to be less than 0.001 (p<0.001). This indicates a high level of significance in line with our predetermined threshold of 0.05. As 0.001<0.05, we reject the null hypothesis and accept the alternative hypothesis. You can refer to the specific changes on page 25, lines 637-649.

**Author Action:**
